# Supplementary material for: Next generation genetically encoded fluorescent sensors for serotonin
Source: Nat Commun. 2022 Dec 6;13:7525. doi: 10.1038/s41467-022-35200-w (PMC9726753; doi:10.1038/s41467-022-35200-w)
Supplement: Supplementary file 1 — Supplementary Information [file 41467_2022_35200_MOESM1_ESM.pdf]

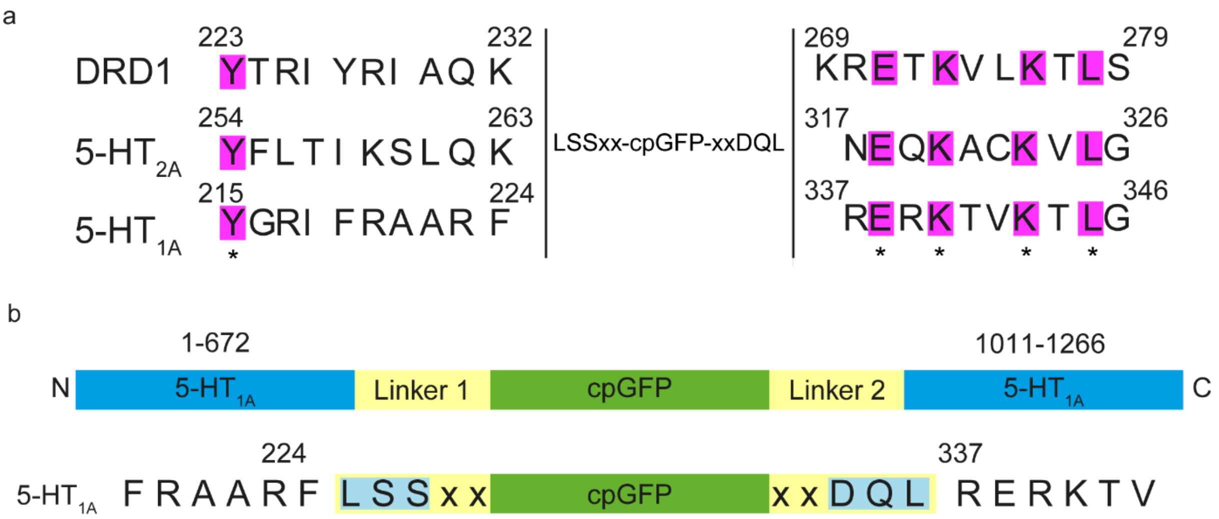

Supplementary figure S1: Design principle of sDarken. a) the third intracellular loop of the 5-HT_{1A} receptor is replaced by cpGFP. Optimization occurred by mutation of linker 1 and 2. In linker 1 amino acids 4 (position 1) and 5 (position 2) are randomly mutated, whereas in linker two amino acids 1 (position 3) and 2 (position 4 have) been mutated. Amino acid abbreviations: A Ala,C Cys, D Asp, E Glu, F Phe, I Ile, K Lys, L Leu, N Asn, Q Gln, R Arg, S Ser, T Thr, V Val, Y Tyr. b) Sequence alignment of DRD_1, 5-HT_{2A} and 5-HT_{1A}

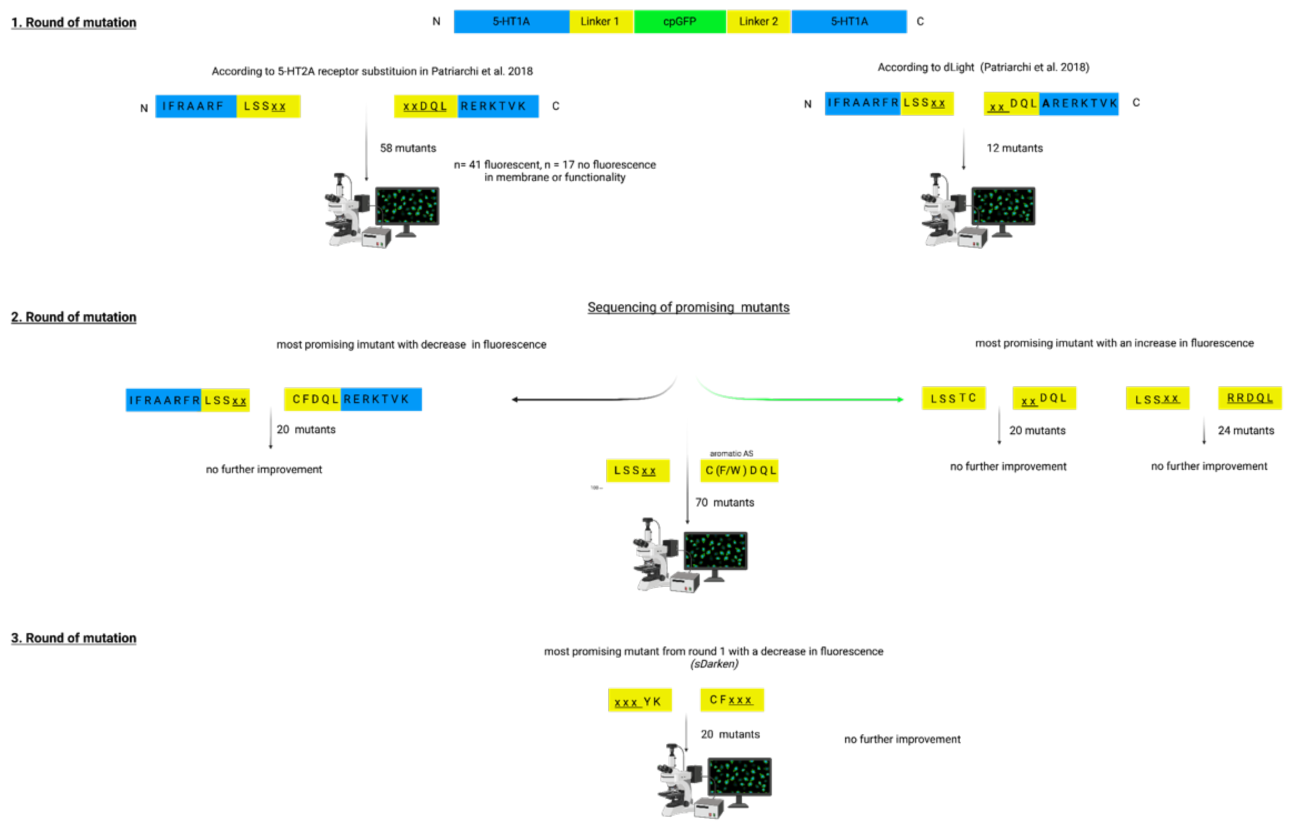

Supplementary figure S2: Library design. In total 224 mutants were expressed and analyzed in HEK cells. In the first round of mutagenesis, in which we followed the design strategies of Patriachi et al. 2018, 58 mutants were designed according to the replacement strategy used for the 5-HT2A in Patriarchi et al. 2018 and 12 additional mutants were generated based on dLight. Out of the 58 replacement mutants only 41 showed membrane fluorescence and functionality. The 12 mutants according to dLight were not different in functionality or expression. No obvious pattern for functionality was evident expect a favor for aromatic amino acids in position 2 and 3 of the C-terminal linker. In a second round of mutations, several strategies were applied in parallel to further increase the number of functional mutants: First, position 4 and 5 in the N-terminal linker 1 were again randomly mutated, while retaining an aromatic amino (F/W) acid in the C-terminal linker at position 2 and 3. 70 of these mutants were randomly chosen and further analyzed. In parallel, we selected the two mutants with an increase in fluorescence and mutated them further in different linker positions. 44 of these were further analyzed but revealed no further improvement in desired properties. In addition, according to Patriarchi et al. 2018, we changed the insertion site of cpGFP and analyzed another 20 mutants, in which the position of the cpGFP was varied. In a third round of mutations we started with the most promising mutant so far (M34) and mutated either position 1, 2, 3, of the N-terminal Linker or position 3, 4, 5 of the C-terminal linker randomly. None of the 20 additional mutants showed any improvement over the original variant (M34). As mutant M34 already showed a high and stable brightness, a large change in signal amplitude upon application of serotonin and superior expression in the membrane, we decided to characterize M34, from now on termed *sDarken*, further. Created with BioRender.com

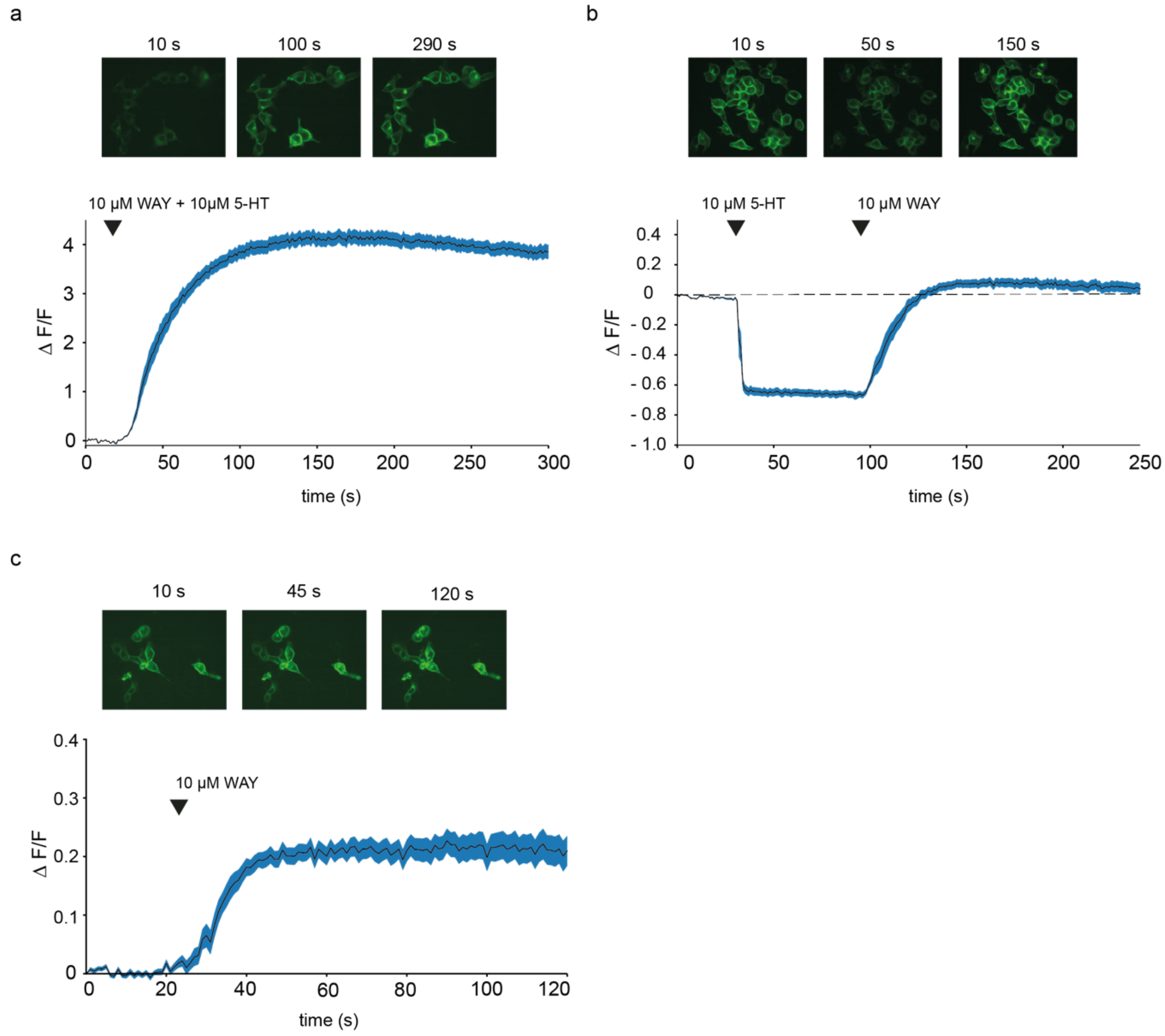

Supplementary figure S3: Response of sDarken to the selective 5-HT$_{1A}$ antagonist WAY 100635. a) Wash in of 10 µM WAY and 10 µM 5-HT at frame 15-60. n=24. b) Application of 10 µM 5-HT followed by wash in of 10 µM WAY, n=12, mean ± SEM. c) Application of WAY 100635 only at frame 30-40, n=23, mean ± SEM.

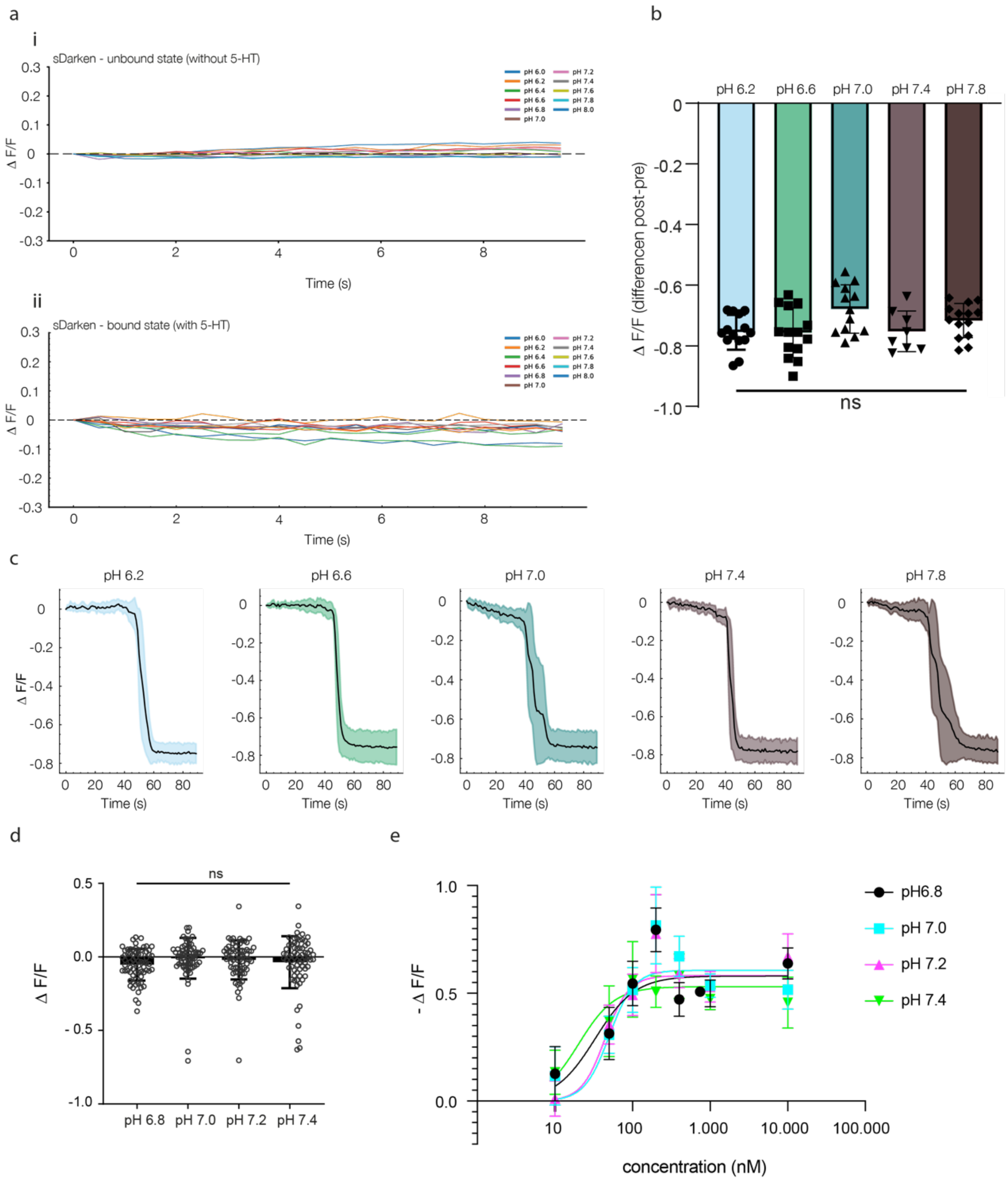

Supplementary figure S4: Influence of different pH values on sDarken fluorescence.
a) Measurement of fluorescence stability over time during different pH values, either without 5-HT (i) or with 5-HT (100 µM) (ii). No apparent effect of the different pH values could be spotted. Individual colored lines represent the mean over time. b) Fluorescence changes due to 5-HT stimulation (post-pre; 5 frame average before and after 5-HT wash in) during different pH values. No significant differences could be detected between the different pH values (Welch's ANOVA, Dunnett's Post Hoc Test; pH 6.2: n=14 cells from 2 trials, pH 6.6: n=14 cells from 2 trials, pH 7.0: n=13 cells from 2 trials, pH 7.4: n=8 cells from 2 trials, pH 7.8: n=14 cells from 2 trials). replicates c) Mean time courses ± standard deviation of the data plotted in b. d) HEK cells expressing *sDarken* did not show any significant fluorescence changes (RM one-way-ANOVA) to Ringer with different pH values as indicated (n= 70 cells, from 14 trials). Fluorescence changes were measured in one consecutive recording with a randomized order of pH values. Normalization (ΔF/F) was performed with the first frame as F0. e) Dose response curve measured in response to different 5-HT concentrations at different pH values (2 trials per concentration and pH value, at least 18 cells per condition). ΔF/F was separately calculated for each pH value. pH 6.8: Kd=33 nM, pH7.0: Kd= 50 nM, pH7.2: Kd = 44 nM, pH7.4: Kd = 20 nM). mean ± SD, n.s. not significant.

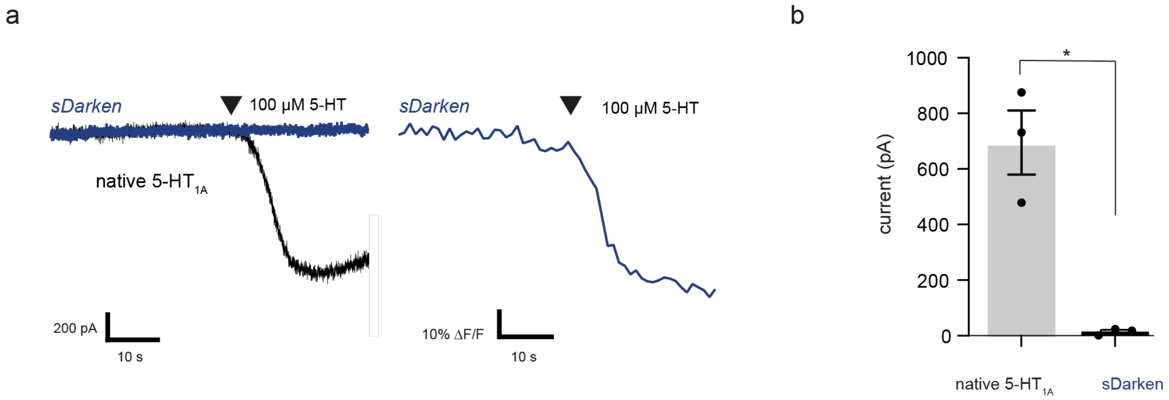

Supplementary figure S5: sDarken has no effect on G_i-signaling. a) Example trace of a GIRK current recording in HEK cells stably expressing GIRK 1 and GIRK 2 subunits. Application of 100 µM 5-HT induced only potassium currents in cells expressing the native 5-HT_{1A} receptor (a, left, black trace), whereas no current response could be observed in HEK cells expressing sDarken (a, left, blue trace). b) Comparison of maximal GIRK current amplitude elicited by the application of 100 µM 5-HT (n=3). Values are given as mean ± SEM. Unpaired two-tailed t-test. p=0.0042, ** $p < 0.01$.

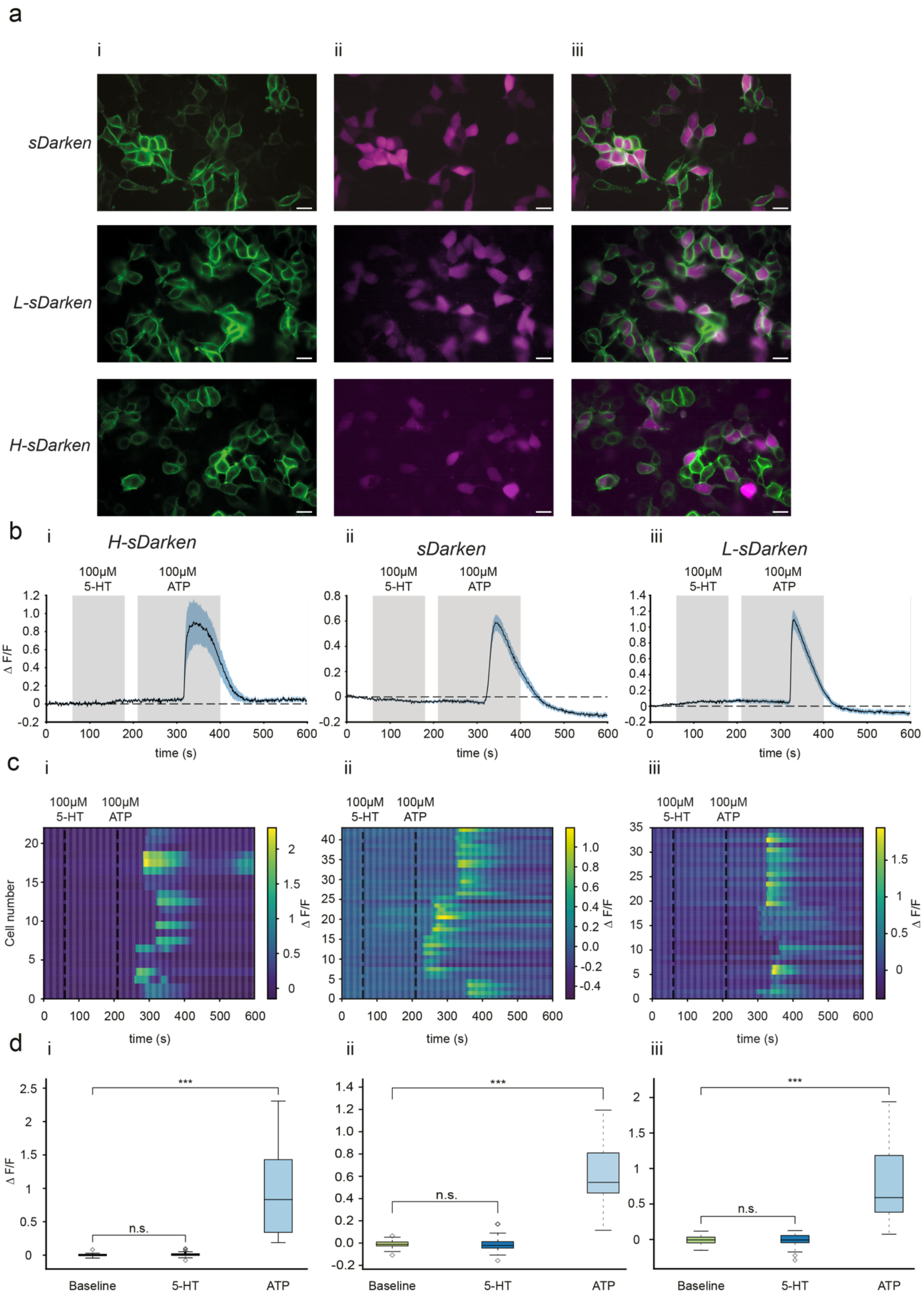
a
i
ii
iii
sDarken
L-sDarken
H-sDarken
b
i
H-sDarken
100µM 5-HT
100µM ATP
Δ F/F
time (s)
ii
sDarken
iii
L-sDarken
c
Cell number
Δ F/F
d
Δ F/F
n.s.
***
Baseline
5-HT
ATP

Supplementary figure S6: sDarken variants have no effect on Gq -signalling.
(a) Expression of sDarken, L-sDarken or H-sDarken and jRCaMP in HEK293T cells, scale bar: 10 µm. i) Fluorescence signal of sDarken, L-sDarken or H-sDarken (green) ii) Fluorescence signal of jRCaMP (magenta). iii) Overlay of sDarken, L-sDarken or H-sDarken and jRCaMP fluorescence signals. (b) Mean ΔF/F values over time (sec) for exemplary trials (i H-sDarken: n=6, ii sDarken: n=17, iii L-sDarken: n=15). Shaded area indicates SEM. Perfusion of cells with 100 µM 5-HT started after 60 seconds and lasted for 120 secs. Subsequently, perfusion of cells with 100 µM ATP started after 220 seconds and lasted for 180 secs. Only cells that co-expressed the 5-HT sensor (sDarken, L-sDarken or H-sDarken) and jRCaMP were included in the analysis. (c) Heatmap of jRCaMP ΔF/F values of all recorded HEK293T cells in all trials (i H-sDarken: n=22, ii sDarken: n= 43 cells, iii L-sDarken: n=35) over time. Dashed lines indicate stimulation of the recorded cells with 100µM 5-HT and subsequently 100 µM ATP. (d) Box-plots of jRCaMP ΔF/F values depicted in (c) before (Timepoint: 10s before 5-HT stimulation), after 100µM 5-HT stimulation (Timepoint: 60s after start of 5-HT stimulation) and after 100µM ATP stimulation (Timepoint: Maximum ΔF/F values after start of ATP stimulation, see Methods). Box represents the 25% percentile to the 75% percentile. The line in the middle of the box represents the median. The upper and lower whisker marks 1.5 times the IQR from the top (and bottom) of the box. All datapoints outside this range are considered outlier and are plotted as individual points. No effect of the 5-HT stimulation on jRCaMP ΔF/F values was observed with any of the three 5-HT sensors (i H-sDarken: baseline: -0.0074 ΔF/F ± 0.00573 vs. 5-HT 0.00677 ΔF/F ± 0.00831 (median ± SEM); p=0.6636 one-way ANOVA repeated measurements, Dunnetts's post-hoc test; ii sDarken: baseline: -0.0109 ΔF/F ± 0.005 vs. 5-HT -0.0213 ΔF/F ± 0.009 (median ± SEM); p>0.999 one-way ANOVA repeated measurements, Dunnetts's post-hoc test, iii L-sDarken: baseline: -0.0064 ΔF/F ± 0.00971 vs. 5-HT -0.00743 ΔF/F ± 0.0162 (median ± SEM); p=0.3772 one-way ANOVA repeated measurements, Dunnetts's post-hoc test). Stimulation with 100µM ATP significantly increased jRCaMP ΔF/F in HEK293T cells co-expressing either of the three 5-HT sensor variants.(i H-sDarken: baseline: -0.0074 ΔF/F ± 0.00573 vs. ATP 0.832 ΔF/F ± 0.141 (median ± SEM); p<0.0001 one-way ANOVA repeated measurements, Dunnetts's post-hoc test; ii sDarken: baseline: -0.0109 ΔF/F ± 0.005 vs. ATP 0.545 ΔF/F ± 0.0403 (median ± SEM); p=0.0001 one-way ANOVA repeated measurements, Dunnetts's post-hoc test; iii L-sDarken: baseline: -0.0064 ΔF/F ± 0.00971 vs ATP 0.590 ΔF/F ± 0.0966 (median ± SEM); ; p<0.0001 one-way ANOVA repeated measurements, Dunnetts's post-hoc test).

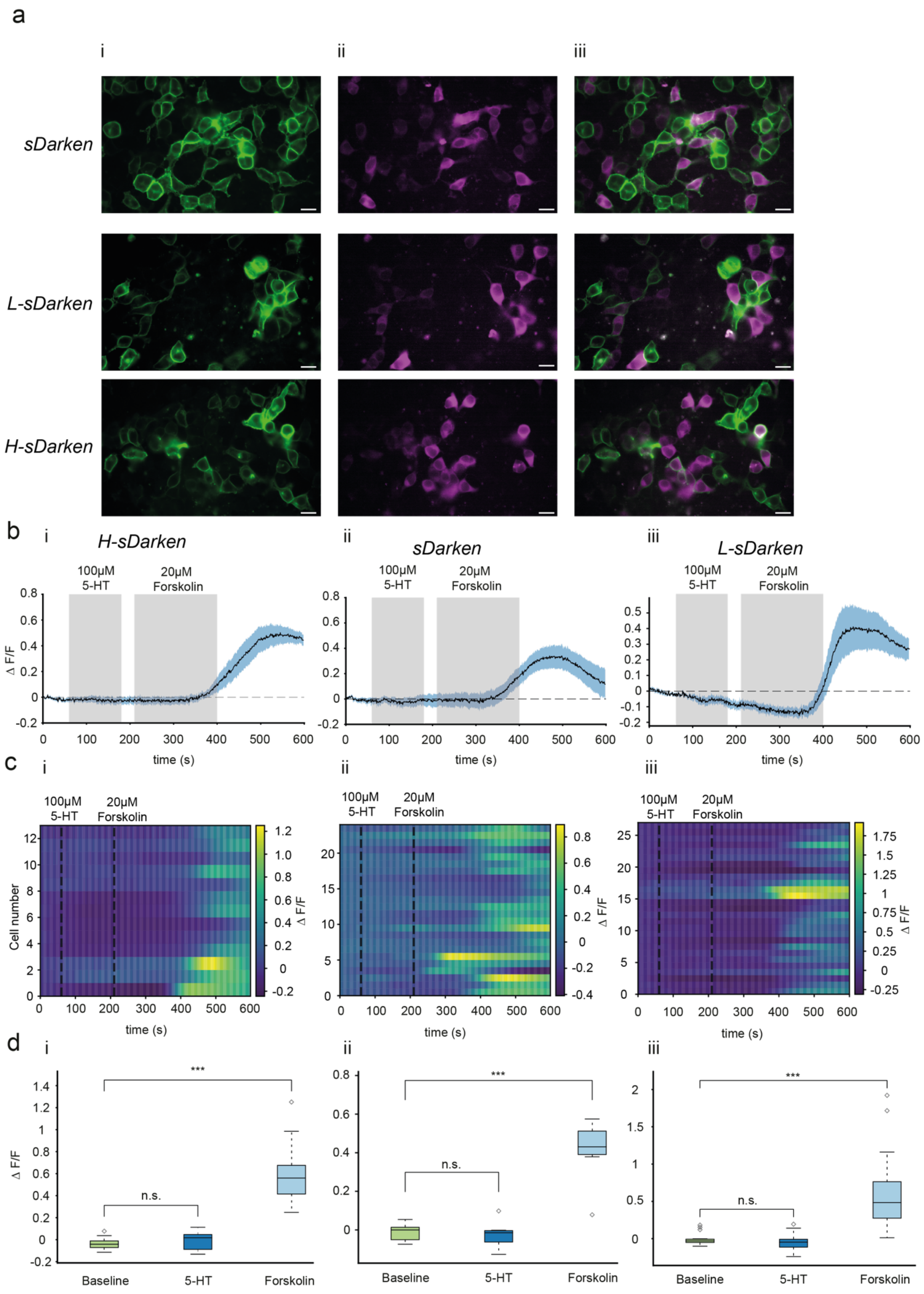

a
i
ii
iii
sDarken
L-sDarken
H-sDarken
b
i
H-sDarken
ii
sDarken
iii
L-sDarken
100μM 5-HT
20μM Forskolin
Δ F/F
time (s)
c
i
ii
iii
Cell number
Δ F/F
time (s)
d
i
ii
iii
Δ F/F
***
n.s.
Baseline
5-HT
Forskolin

Supplementary figure S7: Darken variants have no effect on Gs-signalling.
(a) Expression of sDarken, L-sDarken or H-sDarken and CNG-jRCaMP in HEK cells, scale bar: 10 µm. i) Fluorescence signal of sDarken, L-sDarken or H-sDarken (green) ii) Fluorescence signal of CNG-jRCaMP (magenta). iii) Overlay of sDarken, L-sDarken or H-sDarken and CNG-jRCaMP fluorescence signals. (b) Mean Delta f/f values over time (sec) for exemplary trials (i H-sDarken: n=13, ii sDarken: n=5, iii L-sDarken: n=4). Shaded area indicates SEM. Perfusion of cells with 100µM 5-HT started after 60 seconds and lasted for 120 secs. Subsequently, perfusion of cells with 20µM Forskolin started after 220 seconds and lasted for 180 secs. Only cells that co-expressed the 5-HT sensor (sDarken, L-sDarken or H-sDarken) and CNG-RCaMP were included in the analysis. c) Heatmap of CNG-jRCaMP ΔF/F values of all recorded HEK293T cells in all trials (i H-sDarken: n=13 cells, ii sDarken: n= 24 cells, iii L-sDarken: n=27 cells) over time. Dashed lines indicate stimulation of the recorded cells with 100µM 5-HT and subsequently 20 µM Forskolin. (d) Box-plots of CNG-jRCaMP ΔF/F values depicted in (c) before (Timepoint: 10s before 5-HT stimulation), after 100µM 5-HT stimulation (Timepoint: 60s after start of 5-HT stimulation) and after 20µM Forskolin stimulation (Timepoint: Maximum ΔF/F values after start of Forskolin stimulation, see Methods). Box represents the 25% percentile to the 75% percentile. The line in the middle of the box represents the median. The upper and lower whisker marks 1.5 times the IQR from the top (and bottom) of the box. All datapoints outside this range are considered outlier and are plotted as individual points. No effect of the 5-HT stimulation on CNG-jRCaMP ΔF/F values was observed with any of the three 5-HT sensors (i H-sDarken: baseline: -0.00558 ΔF/F ± 0.00592 vs. 5-HT: 0.00785 ΔF/F ± 0.00871 (median ± SEM); p>0.999 one-way ANOVA repeated measurements, Dunnetts's post-hoc test; ii sDarken: baseline: baseline: -0.0144 ΔF/F ± -0.0313 vs. 5-HT: -0.0313 ΔF/F ± 0.0153 (median ± SEM); p=0.2454 one-way ANOVA repeated measurements, Dunnetts's post-hoc test, iii L-sDarken: baseline: -0.0306 ΔF/F ± 0.0128 vs. 5-HT: -0.0469 ΔF/F ± 0.0181 (median ± SEM); p=0.0678 one-way ANOVA repeated measurements, Dunnetts's post-hoc test). Stimulation with 20µM Forskolin significantly increased CNG-jRCaMP ΔF/F in HEK293T cells co-expressing either of the three 5-HT sensor variants.(i H-sDarken: baseline: -0.00558 ΔF/F ± 0.00592 vs. Forskolin: 0.852 ΔF/F ± 0.147 (median ± SEM); p<0.0001 one-way ANOVA repeated measurements, Dunnetts's post-hoc test; ii sDarken: baseline: -0.0144 ΔF/F ± -0.0313 vs. Forskolin: 0.409 ΔF/F ± 0.0429 (median ± SEM); p=0.0006 one-way ANOVA repeated measurements, Dunnetts's post-hoc test; iii L-sDarken: baseline: -0.0306 ΔF/F ± 0.0128 vs Forskolin 0.484 ΔF/F ± 0.0904 (median ± SEM); p=0.0029 one-way ANOVA repeated measurements, Dunnetts's post-hoc test).

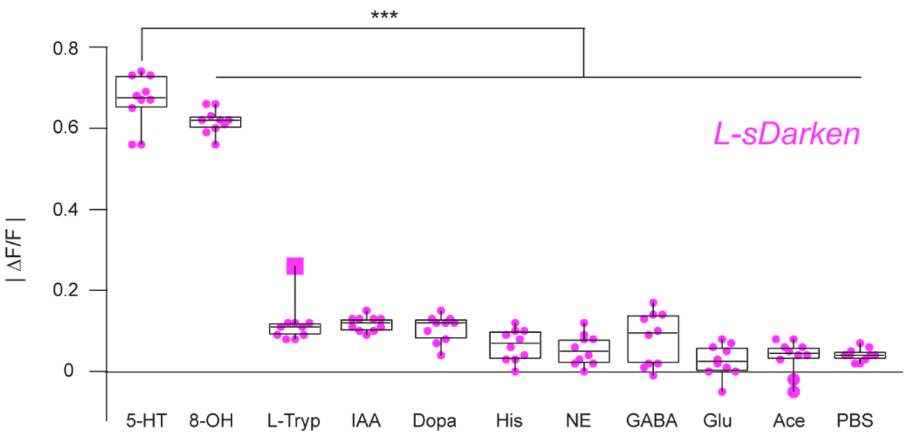

Supplementary figure S8: Specificity of L-sDarken in response to high concentrations

Fluorescence change to application of serotonin (1.6 mM), 8-OHDPAT (300 µM), similar substances or neurotransmitters if not mentioned differently 3 mM were applied (n=10). Box represents the 25% percentile to the 75% percentile. The line in the middle of the box represents the median.Values are given as mean ± SEM. One-Way Anova multiple comparison,*** $p < 0.001$, ** $p < 0.01$. * $p < 0.01$, n.s. not significant

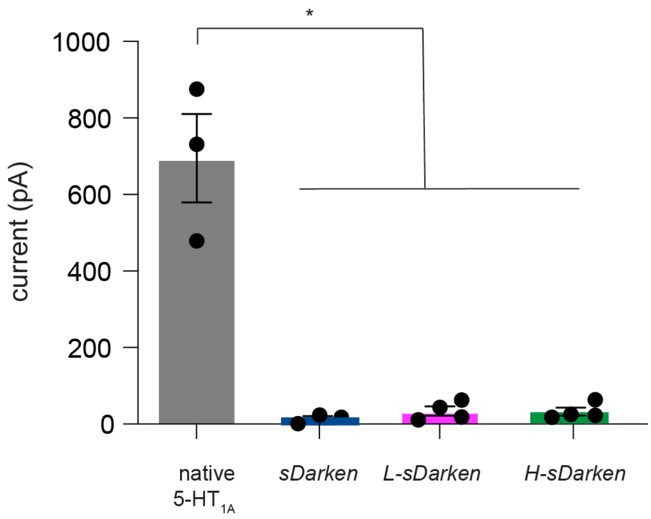

Supplementary figure S9: GIRK current in response to application of 5-HT. Application of 100 µM 5-HT induced only potassium currents in cells expressing the native 5-HT_{1A} receptor, whereas no current response could be observed in sensor expressing HEK cells. Comparison of maximal GIRK current amplitude elicited by the application of 100 µM 5-HT or 1 mM 5-HT for L-sDarken (5-HT_{1A} n=3, sDarken n=3, L-sDarken n=4, H-sDarken n=4. Values are given as mean ± SEM. *** $p < 0.001$, ** $p < 0.01$. * $p < 0.01$, n.s. not significant

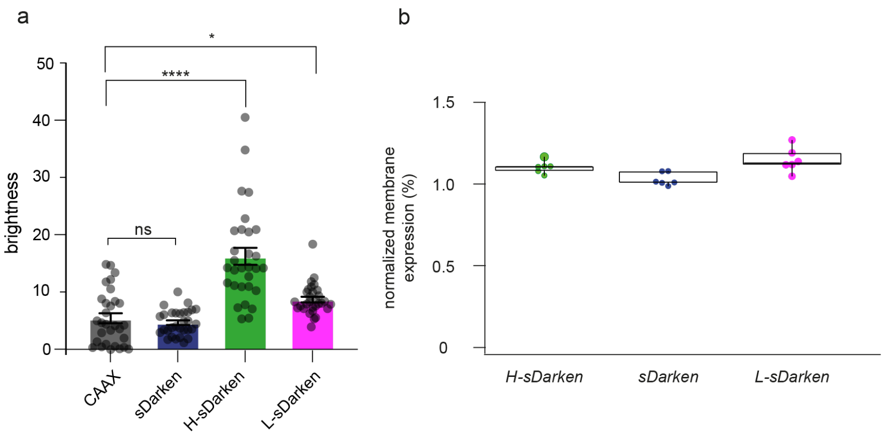

Supplementary figure S10: Brightness and expression of sDarken variants.
a) Fluorescence intensity of CAAX and the different sensor variants (expressed in HEK cells) after background subtraction (n=30 cells each). Significant differences could be detected between CAAX and H-sDarken (p<0.0001, Dunnett's multiple comparisons test) and CAAX and L-sDarken (p=0.0352, Dunnett's multiple comparisons test). b) Membrane expression was calculated as membrane to cytosol fluorescence ratio normalized to CAAX-eGFP (n=6 cells) Box represents the 25% percentile to the 75% percentile. The line in the middle of the box represents the median. (Patriarchi et al. 2018).

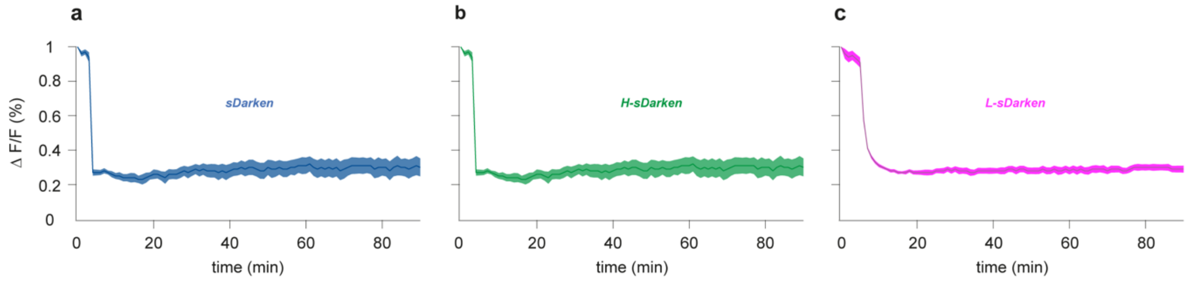

Supplementary figure S11: Long-term exposure to serotonin. Fluorescence response of sensor transfected cells to the application of 5-HT for 90 mins. a) sDarken application of 100 µM 5-HT, n=5 b) H-sDarken application of 100 µM 5-HT, n=5 c) L-sDarken application of 1 mM 5-HT, n=5. mean ± SEM

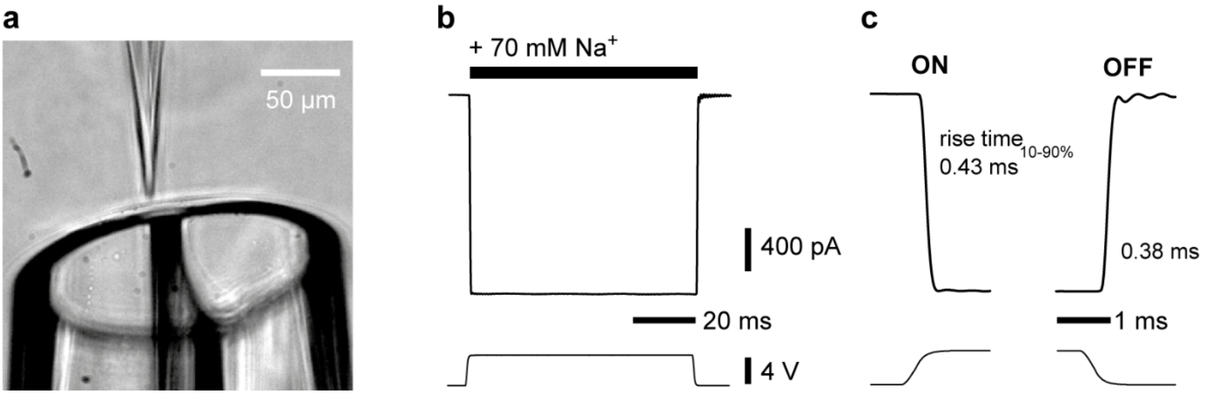

Supplementary figure S12: Fast piezo-driven solution exchange. (a) A patch pipette with an excised outside-out patch (top) is placed in front of a piezo-driven double-barreled glass pipette (bottom). One channel contains ligand, the other channel extracellular solution only. For details on the solution exchange system see Pollok & Reiner, 2020. (b) Measurement of fast solution exchange. Exchange currents were monitored by switching between and 70 mM and 140 mM NaCl (downward deflections) at an open patch pipette in voltage-clamp mode. The filtered voltage step, which drives the piezo element, is shown at the bottom. (c) Details of the ON and OFF phase. The current rise and decay times 10-90% are in the submillisecond range. For details see SI Methods.

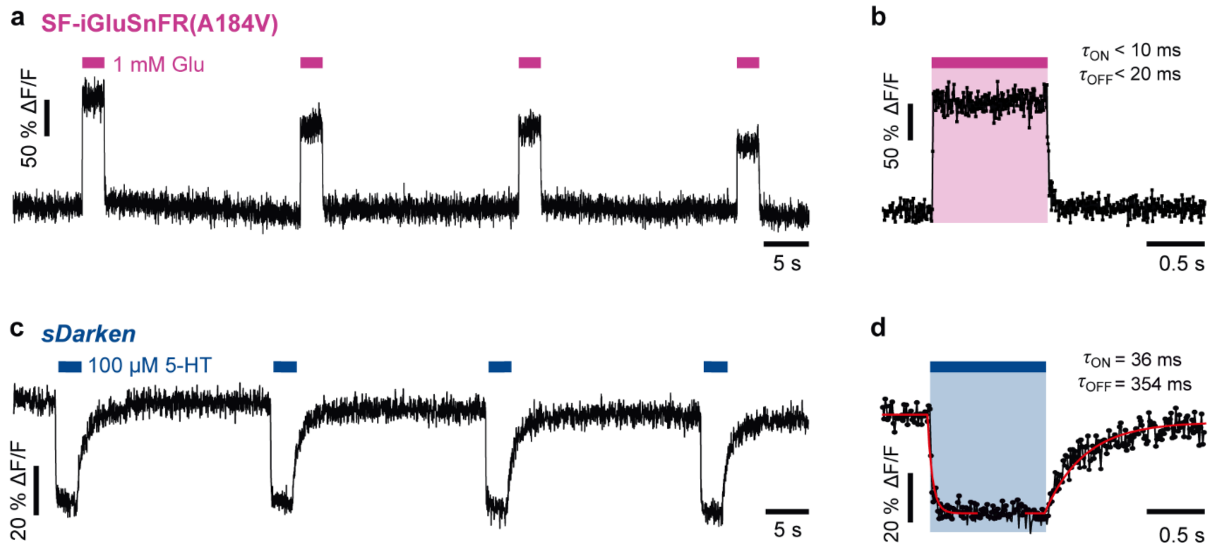

Supplementary figure S13: Comparison to iGluSnFR. (a) Repeated application of 1 mM glutamate to a patch from a HEK cell expressing SF-iGluSnFR(A184V) (Marvin et al. 2018) results in fluorescence increases. (b) The example shows a single sweep with ~160% signal change. The ON/OFF kinetics of SF-iGluSnFR(A184V) are fast compared to imaging (194 fps). For the kinetics of iGluSnFR/SF-iGluSnFR see also Helassa et al. 2018 and Marvin et al. 2018. (c) sDarken ON/OFF responses upon application of 100 µM 5-HT. (d) Single sweep with ~40% signal change. The kinetics are reasonably well described by single exponential fits (red lines; cf. Fig. 4b) with time constants as indicated. For details see SI Methods.

a

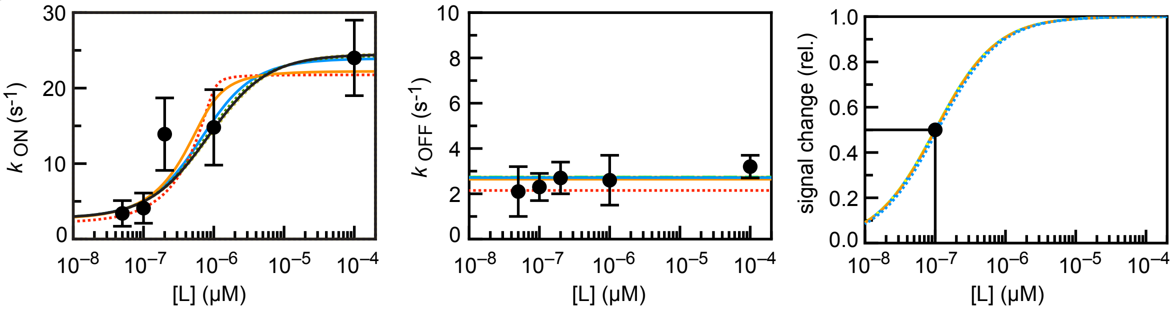

fifit	k_{12} (10^7/(M s))	k_{21} (1/s)	k_{23} (1/s)	k_{32} (1/s)	chi^2	k_{12} / k_{21} (10^6/M)	k_{OFF}^{app} (1/s)	K_d^{app} (10^{-7} M)
	2.00 ^{fix}	2.435	2.315	19.439	10.250	8.21	2.1473	1.879
	3.05 ^{fix}	6.368	11.446	10.773	4.594	4.79	2.6442	1.0122
	**6.109**	**32.540**	**19.439**	**4.488**	**4.122**	**1.88**	**2.7171**	**0.9999**
	12.0 ^{fix}	85.005	20.892	3.424	4.161	1.41	2.7303	0.9999
	24.0 ^{fix}	192.295	21.427	3.045	4.202	1.25	2.7353	0.9999
	100.0 ^{fix}	872.389	21.769	2.807	4.239	1.15	2.7386	0.9965

b

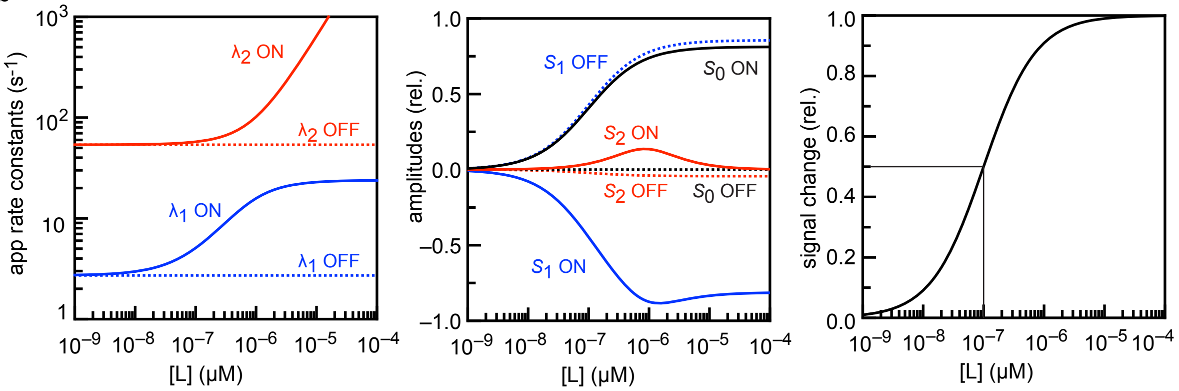

Supplementary figure S14: *sDarken* signal changes are reproduced by a three-state model. (a) Fitting of the model (Eq. 1) to the experimental data k_{ON} (*left*), k_{OFF} (*middle*) and K_D (*right*). The freely fitted parameter set ($k_{12} = 6.1\cdot10^7$ M^{-1} s^{-1}) is shown in blue. Parameters sets obtained by fitting with fixed k_{12} rate constants show similar fit quality, if $k_{12} > 3\cdot10^7$ M^{-1} s^{-1} (for details see SI Note Xkineticmodel). (b) Apparent rate constants λ_1 and λ_2 (*left*), corresponding amplitudes S_0, S_1 and S_2 (*middle)*, and normalized *sDarken* signal change (*right*) as a function of the ligand concentration [L] calculated for the freely fitted rate constants. Under these conditions, the ON kinetics (solid lines) and OFF kinetics (dashed lines) are dominated by S_1, i.e. the experimentally observed signal changes mostly obey single exponential kinetics.

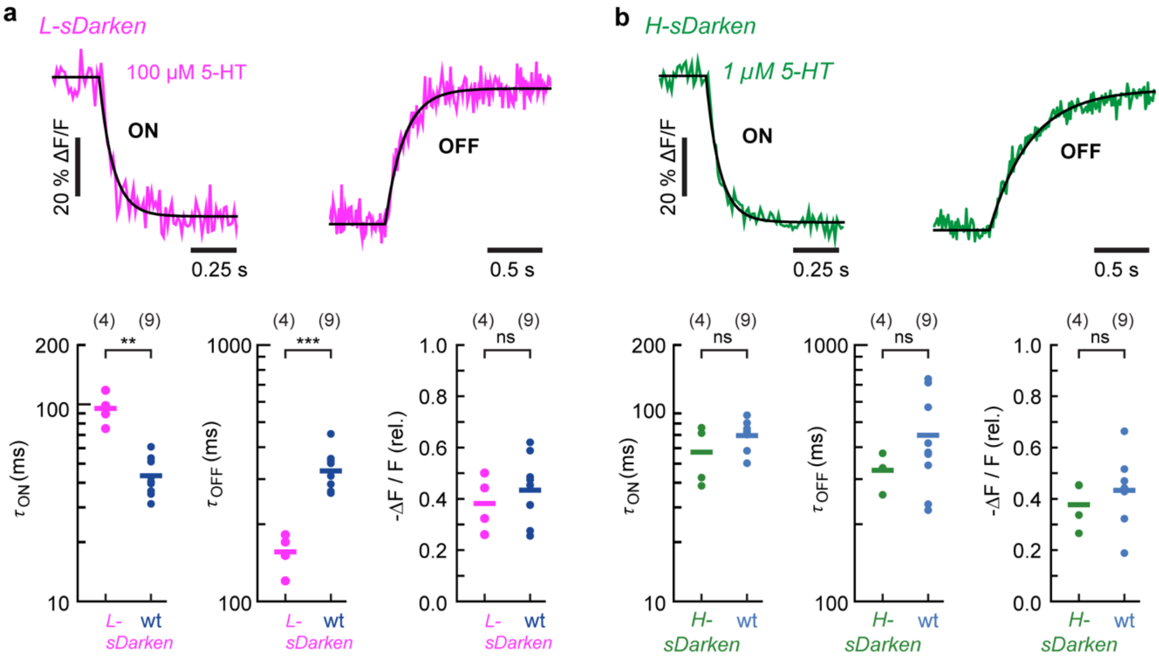

Supplementary figure S15: ON/OFF kinetics of *L-sDarken* and *H-sDarken*. (a) ON/OFF kinetics of L-sDarken (pink) upon application of 100 µM 5-HT Top: Representative trace (average of 4 sweeps) with single exponential fits. Bottom: Quantification shows significantly slower ON and significantly faster OFF kinetics compared to sDarken (wt) at 100 µM 5-HT, but unaltered signal changes (number of patches given in parenthesis, means shown as crosses). (b) ON/OFF kinetics of H-sDarken (green) upon application of 1 µM 5-HT. Top: Representative trace (average of 5 sweeps) with single exponential fits. Bottom: Quantification shows similar ON kinetics, OFF kinetics and signal changes compared to sDarken (wt) at 1 µM 5-HT (number of patches given in parenthesis, means shown as bars). Statistical testing was performed using Welch's t-test, ** $p < 0.01$, *** $p < 0.001$. + boxes

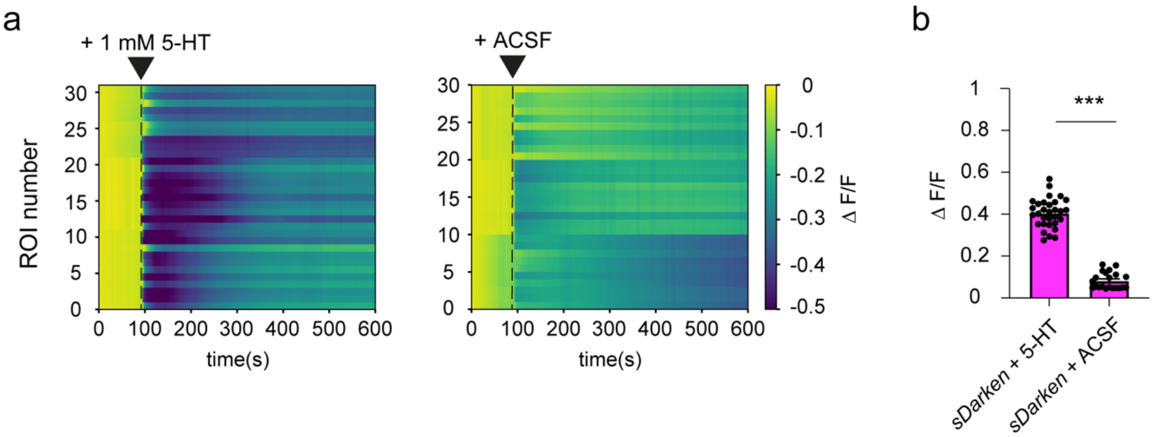

Supplementary figure S16: Puff application in brain slices, that express *L-sDarken* (a) Left panel puff application of 1mM 5-HT, n=30 ROIs from 3 slices. Right panel puff application of ACSF, n=30 ROIs in 3 slices. (b) Quantification of fluorescence decrease after puff application from data in a, L-sDarken +5-HT 0.41±0.01 n=30 ROIs in 3 brain slices, L-sDarken+ ACSF 0.08±0.008, n=20 2 brain slices, unpaired two-tailed t-test, n= mean± SEM *** $p < 0.001$.

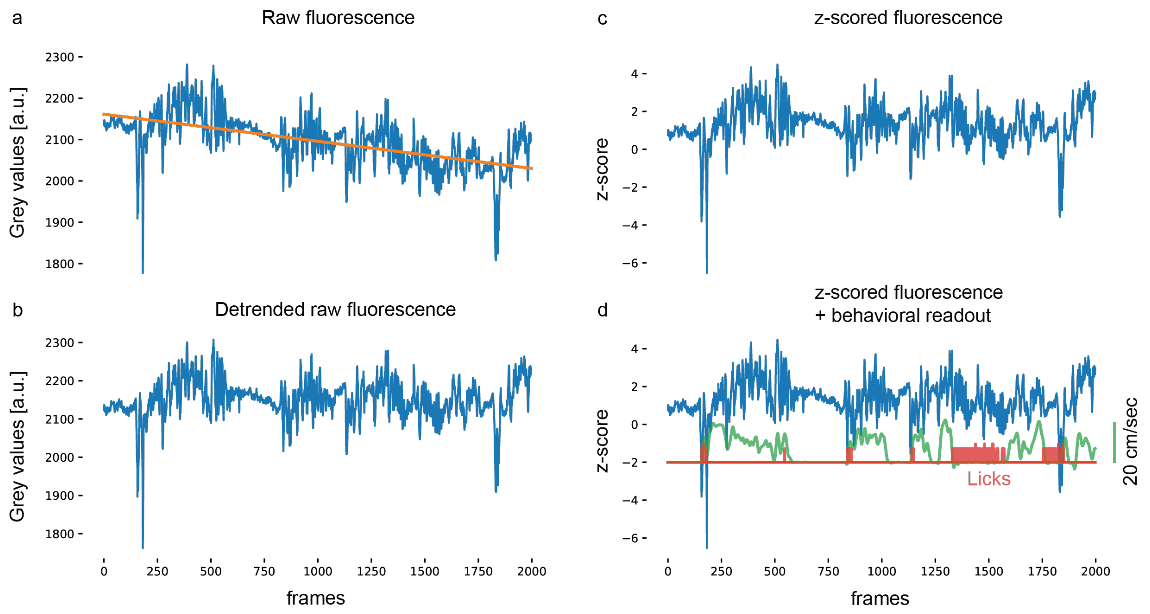

Supplementary figure S17: Data processing steps. (a) Raw fluorescence data of a full field ROI. **(b)** Detrended raw fluorescence based on linear regression. **(c)** Z-scored fluorescence. **(d)** Z-scored fluorescence (blue) plotted together with running speed (green) and licking (red).

Supplementary Materials and Methods

Fast patch-clamp fluorometry

5-HT sensor (sDarken) kinetics were analyzed by fast ligand applications to outside-out membrane patches in combination with fluorescence imaging, i.e. by fast patch-clamp fluorometry.

Sensor expression and patch-clamp procedure.
HEK 293T cells were grown in DMEM with 8 % FBS at 37 °C and 5 % CO_2 on plastic (PET-G) coverslips. Transfections were conducted after 24-48 h using polyethylenimine 25,000 with ~0.3 µg DNA per ml medium. Membrane patches were pulled after 48 h expression using standard patch-clamp procedures. In brief, patch pipettes (3-4 MΩ resistance) were pulled from borosilicate glass (G150TF-4, Warner Instruments) and filled with internal solution (122 mM CsCl, 2 mM NaCl, 2 mM $MgCl_2$, 10 mM EGTA, 10 mM HEPES, pH 7.2). Coverslips with adherent cells were placed in external solution (138 mM NaCl, 1.5 mM KCl, 1.2 mM $MgCl_2$, 2.5 mM $CaCl_2$, 10 mM HEPES, pH 7.3). Using a micromanipulator (Patchstar, Scientifica) and an Axopatch 200B patch-clamp amplifier (in combination with a Digidata 1550 A/D converter and pClamp 10.7 software; all Molecular Devices) cell-attached and then whole-cell configurations were established to excise outside-out patches. Experiments were performed at 22-25 °C with outside-out patches voltage-clamped to -70 mV. The resistance was typically ≥1 GΩ and was monitored throughout the experiment. pClamp and the A/D converter were used for generating the voltage pulses for ligand application and triggering the acquisition of single camera frames. Chemicals, including 5-HT hydrochloride and glutamate, were purchased from Sigma. Control experiments were performed with pAAV SFiGluSnFR.A184V (Addgene #106199) (Marvin et al, 2018).

Fast ligand application and imaging.
Fast ligand application and removal was achieved by positioning the outside-out patches in front of a piezo-driven double-barreled *Θ*-glass pipette (Jonas 1985; Reiner & Isacoff 2014; Pollok & Reiner 2020). The pipette was pulled from borosilicate glass (OD 2.0 mm, ID 1.40 mm, septum 0.2 mm, Warner Instruments), broken to yield a ~150 µm diameter tip, and mounted to a piezo actuator (P842.20, Physik Instrumente). Lateral displacements were triggered with short voltage steps (3 V ramp in 0.7 ms), amplified by a power supply (E505.10, Physik Instrumente) and filtered at 1 kHz. Solutions were delivered using a syringe pump (0.2-0.4 ml/min per channel) with parallel bath perfusion at ~3-5 ml/min. Exchange currents between extracellular solution 0.5 x and extracellular solution 1 x confirmed sub-millisecond solution exchange (**Fig. S12**). Typically, 5-HT was applied for 1 s in 6-8 subsequent sweeps. For lower concentrations the application time was increased up to 4 s.
Epifluorescence imaging was performed on an inverse microscope (DMi8, Leica), with a 40x objective (HCX PL FL L 40x/0.60 CORR XT). Green fluorescence was excited using a 470 nm LED (Thorlabs) and a 470/40 nm excitation filter in combination with a 495 nm dichroic mirror and a 525/50 nm emission filter (all Chroma). The light intensity in the focal plane was ~10.4 mW/mm^2. Images were acquired with an EMCCD camera (Evolve 512 delta, Photometrics) using MicroManager2.0 (Edelstein et al., 2014). The acquisition of individual frames was controlled by TTL pulses (presequence/strobed mode) triggered via pClamp 10.7 (Molecular Devices). Imaging of the 5-HT sensor (**Fig. 2**, **Fig. 3**, **Fig. S13** and **Fig. S14**) was performed with an effective frame rate of 91 Hz using an exposure time of 10 ms, a gain

setting of 50, and cropping to a region of 9 x 16 pixels. SF-iGluSnFR(A184V) (**Fig. S13**) responses were measured with an exposure time of 5 ms at an effective frame rate of 194 Hz.

Data analysis.

Experiments were repeated several times after independent transfections. ImageJ 1.53c (Rasband W./NIH) was used to extract the fluorescence intensity of a defined region (9-to-16 pixel oval) of the membrane patch and a neighboring region for background subtraction. The data were transferred to Clampfit 7 (Molecular Devices), baseline corrected (linear adjustment) and averaged for further analysis. Fluorescence changes, $\Delta F/F$ (%), and time constants from single exponential fits, τ_{ON} and τ_{OFF}, (**Fig. 3b**) were determined from averaged traces (typically 6-8 sweeps) by least-square fitting using ProFit 7.0 (Quantumsoft). Single exponential fits provided a reasonable description of all traces, although a minor slow phase was visible in some cases. These slow phases, typically present in the ON and OFF kinetics, varied between patches and are likely to reflect inhomogeneous perfusion in the patch pipette. Statistical analysis was performed with Excel (Microsoft) and Statistica 13.3 (StatSoft). All data sets showed normal distribution (Shapiro-Wilk tests, $p < 0.05$). One-way ANOVA ($p < 0.05$) followed by a Tukey-Kramer post-hoc testing procedure was used to compare up to five conditions (**Fig. 3b**). Pair-wise comparison was performed using Welch's *t*-test (**Fig. S15**). Calculations on the kinetic model (**Fig. S14** were performed with ProFit 7.0 (Quantumsoft). Fluorescence changes are shown in false colors (**Fig. 2**) (average of 30 frames, baseline corrected).

Supplementary Note

Kinetic model describing the 5-HT sensor

We investigated the concentration dependence of *Darken* (**Fig. 3**). First, the observed ON kinetics became faster with increasing 5-HT concentrations, but in the high concentration range (1-100 µM) the ON kinetics did not increase beyond 15-25 s^{-1}. This indicates that steps other than 5-HT binding become rate limiting for producing the observed signal change (*Darken**, fluorescence decrease). The most simple description of the kinetics can thus be given by a three-state scheme (**Fig. 3** and **Eq. 1**), where the 5-HT binding/unbinding equilibrium (k_{12}, k_{21}) is followed by conformational changes (k_{23}, k_{32}), which result in a reduced cpGFP fluorescence.

$$\text{L + Darken} \underset{k_{21}}{\overset{k_{12}\cdot[\text{L}]}{\rightleftharpoons}} \text{L•Darken} \underset{k_{32}}{\overset{k_{23}}{\rightleftharpoons}} \text{L•Darken}^* \qquad \text{Eq. 1a}$$

$$\text{A} \underset{k_{21}}{\overset{k_{12}'}{\rightleftharpoons}} \text{B} \underset{k_{32}}{\overset{k_{23}}{\rightleftharpoons}} \text{C}^* \qquad \text{Eq. 1b}$$

For binding, pseudo-first order reaction kinetics can be assumed ([L] >> [sensor]), i.e. the rate constant k_{12}' is given by $k_{12}\cdot$[L]. This kinetic scheme predicts double exponential signal changes with two apparent rate constants, λ_1 and λ_2 (**Eq. 2**).

$$S(t) = S_0 + S_1\cdot\exp(-\lambda_1\cdot t) + S_2\cdot\exp(-\lambda_2\cdot t) \qquad \text{Eq. 2a}$$

The corresponding amplitudes $S_{0,}$ S_1 and S_2 associated with 'active' sensor configuration (*Darken**;C) can be obtained analytically for different concentrations, [L], and starting conditions (ON: A_{t0}=1, B_{t0}=C_{t0}=0; OFF: A_{t0}=A_{eq}, B_{t0}=B_{eq}, C_{t0}=C_{eq}) (**Fig. S14**) (Ikai, 1971).

Experimentally single exponential kinetics were observed, which indicates that the second phase ($\lambda_{2,}$ S_2) was associated with small amplitudes and/or negative amplitudes with fast kinetics (fast lag phase). This model can reproduce the experimental observations quite well and fitting with k_{ON}, k_{OFF} and $K_d = 10^{-7}$ M yields: $k_{12} = 6.1 \cdot 10^7$ M^{-1} s^{-1}, $k_{21} = 32.5$ s^{-1}, $k_{23} = 19.4$ s^{-1} and $k_{32} = 4.1$ s^{-1} (**Fig. S14a**). The apparent rate constants and amplitudes resulting for these values are shown in **Fig. S14b**. However, it should be noted that these fits remain poorly defined, as k_{12} (and subsequently k_{21}) can be increased by order of magnitudes without strongly impacting the quality of neither the fits nor the other parameters (**Fig. S14a**). Nevertheless, we find that k_{12} is generally 1-2·10^6 larger than k_{21} and that reasonable fits are only obtained with $k_{12} > 3 \cdot 10^7$ M^{-1} s^{-1} (**Fig. S14a**). In summary, the binding step is fast and already associated with high affinity (0.5-1 µM), whereas the subsequent slower conformational changes appear to be limiting for the observed sensor kinetics.

Supplementary References

Ikai A. and Tanford C. Kinetics of unfolding and refolding of proteins. I. Mathematical analysis. 73, 145–163 (1973).

Jonas P. Fast application of agonists to isolated membrane patches" in Single-channel Recording, B. Sakmann, E. Neher, Eds. ,pp. 231-243(1995).

Pollok S. and Reiner A. Subunit-selective iGluR antagonists can potentiate heteromeric receptor responses by blocking desensitization. Proc Natl Acad Sci U S A., 117(41):25851-25858 (2020).

Reiner A and Isacoff EY. Tethered ligands reveal glutamate receptor desensitization depends on subunit occupancy. Nat Chem Biol. 10(4): 273-80 (2014).

Edelstein A.D. et al. Advanced methods of microscope control using µManager software. Journal of Biological Methods 1(2):e11 doi:10.14440/jbm.2014.36 (2014).

Marvin, J.S. et al. Stability, affinity, and chromatic variants of the glutamate sensor iGluSnFR. Nature Methods, 15(11), 936–939 (2018).
