# Supplementary material for: Next generation genetically encoded fluorescent sensors for serotonin
Source: Nat Commun. 2022 Dec 6;13:7525. doi: 10.1038/s41467-022-35200-w (PMC9726753; doi:10.1038/s41467-022-35200-w)
Supplement: Supplementary file 3 — Description of Additional Supplementary Files [file 41467_2022_35200_MOESM3_ESM.pdf]

File name: Supplementary Movie 1

Description: Fluorescence change of sDarken in HEK cells Fluorescence decreases upon application of 800nM 5-HT (single application as indicated in the movie). The movie was acquired with 1 fps and plays in fast forward. Realtime (120 s).

File name: Supplementary Movie 2

Description: Fluorescence change of sDarken in HEK cells Fluorescence decreases upon application of 5µM 5-HT (repetitive application as indicated in the movie). The movie was acquired with 1 fps and plays in fast forward. Realtime (120 s).

File name: Supplementary Movie 3

Description: Fluorescence change of L-sDarken in HEK cells. Fluorescence decreases upon rapid application of 1 µM 5-HT (single application as indicated in the movie). The movie was acquired with 1 fps and plays in fast forward real time (5 s).

File name: Supplementary Movie 4

Description: Fluorescence change of L-sDarken in HEK cells. Fluorescence decreases upon rapid application of 200 µM 5-HT (single application as indicated in the movie). The movie was acquired with 1 fps and plays in fast forward real time (5 s).

File name: Supplementary Movie 5

Description: Fluorescence change of L-sDarken in HEK cells. Fluorescence decrease upon rapid application of 1600 µM 5-HT (single application as indicated in the movie). The movie was acquired with 91 fps and plays in real time (5 s).

File name: Supplementary Movie 6

Description: Fluorescence change of H-sDarken in HEK cells. Fluorescence decrease upon rapid application of 50nM 5-HT (single application as indicated in the movie). The movie was acquired with 91 fps and plays in real time (5 s).

File name: Supplementary Movie 7

Description: Fluorescence change of H-sDarken in HEK cells. Fluorescence decrease upon rapid application of 800nM 5-HT (single application as indicated in the movie). The movie was acquired with 91 fps and plays in real time (5 s).

File name: Supplementary Movie 8

Description: Mild Bleaching of tddimer2 under continuous two-photon excitation for 1 min. For details see Fig. 2.

File name: Supplementary Movie 9

Description: No bleaching of sDarken under continuous two-photon excitation for 1 min. For details see Fig. 2.

File name: Supplementary Movie 10

Description: Fluorescence change of sDarken in an outside-out patch. Fluorescence decrease upon rapid application of 100 µM 5-HT (single application as indicated in the movie). The movie was acquired with 91 fps and plays in real time (5 s). For details see Fig. 2 and SI Methods.

File name: Supplementary Movie 11

Description: No change in fluorescence of sDarken in control condition in organotypic hippocampal slice culture.

File name: Supplementary Movie 12

Description: Change in fluorescence of sDarken due to application of 10µM serotonin in organotypic hippocampal slice culture.

File name: Supplementary Movie 13

Description: Example 2P in vivo recording in cortical layer 2/3 upon electrical stimulation in the DR.

File name: Supplementary Movie 14

Description: Example 2P in vivo for recording that shows local fluorescence changes preceding a reward.
