# Supplementary material for: Next generation genetically encoded fluorescent sensors for serotonin
Source: Nat Commun. 2022 Dec 6;13:7525. doi: 10.1038/s41467-022-35200-w (PMC9726753; doi:10.1038/s41467-022-35200-w)
Supplement: Supplementary file 18 — Reporting Summary [file 41467_2022_35200_MOESM18_ESM.pdf]

nature research

Corresponding author(s): Olivia Masseck

Last updated by author(s): Feb 1, 2022

Reporting Summary

Nature Research wishes to improve the reproducibility of the work that we publish. This form provides structure for consistency and transparency in reporting. For further information on Nature Research policies, see our Editorial Policies and the Editorial Policy Checklist.

Statistics

For all statistical analyses, confirm that the following items are present in the figure legend, table legend, main text, or Methods section.

n/a	Confirmed	
☐	☒	The exact sample size (*n*) for each experimental group/condition, given as a discrete number and unit of measurement
☐	☒	A statement on whether measurements were taken from distinct samples or whether the same sample was measured repeatedly
☐	☒	The statistical test(s) used AND whether they are one- or two-sided<br>*Only common tests should be described solely by name; describe more complex techniques in the Methods section.*
☒	☐	A description of all covariates tested
☐	☒	A description of any assumptions or corrections, such as tests of normality and adjustment for multiple comparisons
☐	☒	A full description of the statistical parameters including central tendency (e.g. means) or other basic estimates (e.g. regression coefficient) AND variation (e.g. standard deviation) or associated estimates of uncertainty (e.g. confidence intervals)
☐	☒	For null hypothesis testing, the test statistic (e.g. *F*, *t*, *r*) with confidence intervals, effect sizes, degrees of freedom and *P* value noted<br>*Give P values as exact values whenever suitable.*
☒	☐	For Bayesian analysis, information on the choice of priors and Markov chain Monte Carlo settings
☒	☐	For hierarchical and complex designs, identification of the appropriate level for tests and full reporting of outcomes
☒	☐	Estimates of effect sizes (e.g. Cohen's *d*, Pearson's *r*), indicating how they were calculated

Our web collection on statistics for biologists contains articles on many of the points above.

Software and code

Policy information about availability of computer code

Data collection	For Imaging in HEK cells and brain slices: LNscope from Luigs&Neumann equipped with a CMOS camera (Hamamatsu), Fast ligand application: DMi8 Leica with EMCCD camera (Evolve 512 delta, Photometrics), Confocal Microscopy LSM880 from Zeiss, 2P Imaging in culture: custom built Olympus BX51WI microscope controlled by modified version of ScanImage 3.8 58. Two tunable, pulsed Ti:Sapphire laser (MaiTai DeepSee, Spectra Physics), 2P Imaging in vivo:For acquisition a custom-made Thorlaps two-photon microscope connected with a titanium sapphire 80 MHz Cameleon Ultra II two-photon lase (Coherent, Inc.) and equipped with an 8 kHz galvo-resonant scanner (LSK.GR08/M, Thorlabs), a GaAsP PMT (Thorlabs) and a 16x water immersion objective (Nikon) was used.<br>Whole-cell patch clamp recordings of HEK293 cells: EPC10 USB amplifier (HEKA). Patch Clamp procedure for fast applications:Using a micro-manipulator (Patchstar, Scientifica) and an Axopatch 200B patch-clamp amplifier (in combination with a Digidata 1550 A/D converter and pClamp 10.7 software; all Molecular Devices) were used.
Data analysis	ImageJ (Schneider et al.2012), IgorPro8 (WaveMetrics), ProfFit 7.0 (QuantumSoft)GraphPadPrism 9.3.1.Selfwritten Data analysis in Python will be made availbale upon request.

For manuscripts utilizing custom algorithms or software that are central to the research but not yet described in published literature, software must be made available to editors and reviewers. We strongly encourage code deposition in a community repository (e.g. GitHub). See the Nature Research guidelines for submitting code & software for further information.

Data

Policy information about availability of data

All manuscripts must include a data availability statement. This statement should provide the following information, where applicable:

- Accession codes, unique identifiers, or web links for publicly available datasets
- A list of figures that have associated raw data
- A description of any restrictions on data availability

Data are availbale upon request to the corresponding author.

Field-specific reporting

Please select the one below that is the best fit for your research. If you are not sure, read the appropriate sections before making your selection.

☒ Life sciences ☐ Behavioural & social sciences ☐ Ecological, evolutionary & environmental sciences

For a reference copy of the document with all sections, see nature.com/documents/nr-reporting-summary-flat.pdf

Life sciences study design

All studies must disclose on these points even when the disclosure is negative.

Sample size	Sample size was not predetermined. Sampel size is given for each experiment. Sample size was choosen according to the literature and other studies in the field.
Data exclusions	No data have been excluded from the analysis.
Replication	Each of the described experiments was replicated at least with two replicates.
Randomization	Mice were randomly assigned in experimental groups. Mcie of both sexes were used.
Blinding	Investigators were not blinded to the data or experimental animals.Blinding is usually not performed in the types of experiments that we included.

Reporting for specific materials, systems and methods

We require information from authors about some types of materials, experimental systems and methods used in many studies. Here, indicate whether each material, system or method listed is relevant to your study. If you are not sure if a list item applies to your research, read the appropriate section before selecting a response.

Materials & experimental systems

n/a	Involved in the study	
☒	☐	Antibodies
☐	☒	Eukaryotic cell lines
☒	☐	Palaeontology and archaeology
☐	☒	Animals and other organisms
☒	☐	Human research participants
☒	☐	Clinical data
☒	☐	Dual use research of concern

Methods

n/a	Involved in the study	
☒	☐	ChIP-seq
☒	☐	Flow cytometry
☒	☐	MRI-based neuroimaging

Eukaryotic cell lines

Policy information about cell lines

Cell line source(s)	HEK293T (DSMZ ACC-635), HEK293T+GIRK1/2 (Dr. Tinker, UCL, London)), HEK293T (Sigma Aldrich 12022001)
Authentication	none of the cell lines were authentificated
Mycoplasma contamination	Cells were not tested for Mycoplasma contamination
Commonly misidentified lines (See ICLAC register)	no commonly misidentified lines were used.

Animals and other organisms

Policy information about studies involving animals; ARRIVE guidelines recommended for reporting animal research

Laboratory animals	C57BL/6J mice of both sexes that were 2-6 month old.
Wild animals	the study did not involve wild animals
Field-collected samples	the study did not involve samples form the field
Ethics oversight	Animal protocols were approved by local authorities . Freie Hansestadt Bremen. Senatorin für Gesundheit, Frauen udn Verbraucherschutz or LANUV (Landesamt für Naturschutz und Verbraucherschutz Nordrhein-Westfalen) Germany

Note that full information on the approval of the study protocol must also be provided in the manuscript.
